# Structural and Magnetic Properties of FePd Thin Film Synthesized by Electrodeposition Method

**DOI:** 10.3390/ma13061454

**Published:** 2020-03-23

**Authors:** Gabriele Barrera, Federico Scaglione, Matteo Cialone, Federica Celegato, Marco Coïsson, Paola Rizzi, Paola Tiberto

**Affiliations:** 1 Istituto Nazionale di Ricerca Metrologica (INRiM), Advanced Materials Metrology and Life Sciences, Strada delle Cacce 91, I-10135 Torino, Italy; f.celegato@inrim.it (F.C.); m.coisson@inrim.it (M.C.); p.tiberto@inrim.it (P.T.); 2 Dipartimento di Chimica e Centro Interdipartimentale NIS (Nanostructured Surfaces and Interfaces), University of Turin, Via Pietro Giuria 7, I-10125 Torino, Italy; federico.scaglione@unito.it (F.S.); matteo.cialone@uab.cat (M.C.); paola.rizzi@unito.it (P.R.); 3 Departament de Física, Universitat Autònoma de Barcelona, 08193 Cerdanyola del Vallès, Spain

**Keywords:** FePd alloy, electrodeposition technique, magnetic properties, structural characterisation

## Abstract

Bimetallic nanomaterials in the form of thin film constituted by magnetic and noble elements show promising properties in different application fields such as catalysts and magnetic driven applications. In order to tailor the chemical and physical properties of these alloys to meet the applications requirements, it is of great importance scientific interest to study the interplay between properties and morphology, surface properties, microstructure, spatial confinement and magnetic features. In this manuscript, FePd thin films are prepared by electrodeposition which is a versatile and widely used technique. Compositional, morphological, surface and magnetic properties are described as a function of deposition time (i.e., film thickness). Chemical etching in hydrochloric acid was used to enhance the surface roughness and help decoupling crystalline grains with direct consequences on to the magnetic properties. X-ray diffraction, SEM/AFM images, contact angle and magnetic measurements have been carried out with the aim of providing a comprehensive characterisation of the fundamental properties of these bimetallic thin films.

## 1. Introduction

Bimetallic nanomaterials, composed by a noble element and a magnetic metal, have attracted a growing interest in the scientific community because they show together the properties of both metals in addition to confinement effects [1,2,3,4]. Noble metals play a central role in the area of catalysis [5,6]; whereas the magnetic metal elements (Fe, Co, Ni) with specific magnetic properties offer the possibility to easily recover and reuse the bimetallic nanostructured catalysts after the completion of the reaction and/or the chemical processes [7,8]. In particular, the magnetic separation process provides a practical and useful technique for recycling the magnetic nanostructured catalysts [7,8].

Additionally, the magnetic–noble bimetallic nanomaterials exhibit not only a simple combination of the properties associated with their single elements but they perform enhanced catalyst activity, modified catalyst selectivity or improved catalyst stability with respect to the monometallic catalysts: Sun et al. [9] reveal that the addition of Fe significantly enhances the hydrodeoxygenation activity of Pd/C; Zhao and Gorte [10] found that the activity of Pd/ceria catalysts increases by as much as an order of magnitude upon the addition of a controlled amount of Fe for the water-gas shift; Mu at al. [11] demonstrated that the synergistic effect between Pt and Ni enhances the activity to CO oxidation; additional enhanced catalyst activity by means of magnetic–noble nanomaterials can be found in literature [12,13,14,15,16].

In view of their applications, magnetic–noble bimetallic nanomaterials should be produced in large quantities and at low costs. While nanoparticles may relatively comply well to these constraints, other forms of nanomaterials, such as thin films, could be more difficult to adapt, as their preparation techniques often require large and complex setups with well-controlled vacuum conditions. Electrodeposition technique helps overcoming some of these constraints, being a relatively low-cost and scalable approach for growing metallic thin films, even in the form of bimetallic alloys [17].

Within this context, it is of great importance and scientific interest to design and develop new multifunctional magnetic–noble bimetallic nanomaterials and to study the interplay between the properties and their microstructure, synthesis technique or spatial confinement, as the multiple available degrees of freedom could be exploited to tailor the properties to the desired application [2,15,18,19,20]. In the case of thin films, both bulk (e.g., grain size) and surface (e.g., roughness, surface-to-volume ratio) characteristics turn out to play fundamental roles in determining the resulting properties of the nanomaterial and to define the catalytic activity, therefore an adequate control during the growth process or the subsequent chemical etching is strictly required [21,22].

In this manuscript, we deal with this large subject by focussing on bimetallic thin films combining the noble metal, Pd, with the magnetic transition metal, Fe [23,24,25,26,27]. The former is attracting a wide interest for its catalytic properties, whereas the second, among the magnetic ones, is the least expensive. The FePd bimetallic nanomaterials are prepared in thin films form by electrodeposition, which allows obtaining a Fe-rich composition, and offers the possibility to easily control the film thickness by simply picking the desired deposition time. The surface (e.g., roughness) and magnetic properties depend on the film thickness, as specific microstructure and morphological features develop as a function of deposition time. The Fe-rich composition of the alloy combined with the higher removal rate of the Fe atoms than the one of the Pd atoms through chemical etching in hydrochloric acid allow to refine the roughness on the thickest films and to help the decoupling their grains. Such changes resulted in the ability to fine tune the magnetic properties and increase the surface-to-volume ratio. X-ray diffraction, as well as roughness, contact angle and magnetic measurements have been carried out with the aim of providing a comprehensive characterisation of the fundamental properties of these magnetic–noble bimetallic thin film.

## 2. Materials and Methods

Binary FePd alloy was potentiostatically electrodeposited in a cylindrical electrochemical cell [28], in which a thin foil of platinum acts as counter electrode, a conductive substrate as working electrode and an Ag|AgCl electrode as a reference electrode (V_Ag|AgCl_ = + 0.239 V vs. standard hydrogen electrode at 25 °C). The reference electrode and the working one were mounted into the electrochemical cell in a horizontal arrangement. The depositions were performed at room temperature without stirring the solution. According to the previous work of Konczak et al. [29], the electrolyte composition is 0.01 M Pd(NH_3_)_4_Cl_2_, 0.06 M sulfosalicylic acid (SSA), 0.05 M Fe_2_(SO_4_)_3_ 7H_2_O, 0.3 M (NH_4_)_2_SO_4_ (pH = 5). The magnetic FePd thin film was deposited on the working electrode constituted by a Si/SiO_2_ substrate made conductive by the deposition of an Au layer. The adhesion of Au layer on SiO_2_ surface was ensured by a thin Ti layer deposited between them. Prior to the FePd electrodeposition, the substrates were cleaned consecutively with acetone, isopropanol and de-ionized water in an ultrasonic bath.

The deposition potential was fixed at E_0_ = −1.2 V vs. V_Ag|AgCl_ selecting three different deposition times: 500, 150 and 50 s; the three electrodeposited FePd thin film samples were labelled S500, S150 and S50, respectively.

Scanning Electron Microscopy (SEM) was used to study the morphology of the electrodeposited samples. A software analysis of SEM images enables to calculate the size distribution of the grains. SEM was equipped by energy dispersive x-ray spectrometer (EDS) in order to evaluate the stoichiometry of the FePd alloy.

Thickness and surface morphology of all samples were characterised by means of Atomic Force Microscopy (AFM) operating in intermittent contact mode with the oscillation frequency and amplitude set point achieved by the tuning of the cantilever.

The film thickness was evaluated measuring the height of a well-defined step obtained by a lithographic process between the FePd film and the underlying substrate, see Figure S2 in the Supplementary Materials; the measured thickness was 51, 400 and 631 nm for the S50, S150 and S500 sample, respectively.

From 2D AFM profiles, roughness parameters are calculated such as the root mean square roughness (R_q_), the mean slope of the roughness motifs (1/K_r_) and the steepness of the sample surface (R_q_/ξ) [30,31,32,33,34,35]. The 1/K_r_ parameter is defined as: $K_{r}=\mathrm{AR}/R$, where R is the mean depth of the roughness motifs and AR is the mean spacing of the roughness motifs [30]; whereas the ξ parameter is the correlation length that reflects the average distance between consecutive peaks on the surface [34].

In the manuscript, roughness (R_q_) is reported as the average value and standard deviation of several measurements in different points of the sample surface.

The structure of the as deposited FePd thin films was investigated by grazing angle X-ray diffraction (XRD) with a Cu-K_α_ radiation. The incident X-ray beam was set at a constant grazing angle of 0.6° for all measurements. The moving detector, instead, was rotated around the sample with a step size of 0.02 degrees and an acquisition time of 40 s per step, which were found to provide a good signal-to-noise ratio. A substrate pattern got before electrodeposition was collected and used as a reference in the reflections assignment.

Wettability properties of the samples surface were investigated by means of contact angle measurements performed at room temperature in which a droplet, with a volume of approximately 1 μL, is released from the tip of a calibrated micropipette on the sample surface. Each contact angle value is calculated as the average of at least six measurements in different points of the sample surface.

Magnetic measurements were performed using an Alternating Gradient Field Magnetometer (AGFM) working in the field interval −18 kOe ≤ H ≤ 18 kOe in the parallel configuration (applied magnetic field in the sample plane). Hysteresis loops were obtained at room temperature for all samples and the diamagnetic signal of sample holder and film substrate was adequately subtracted.

First Order Reversal Curves (FORC) [36,37,38] have been measured with the same AGFM instrument, with the aim of identifying reversible and irreversible magnetisation reversal processes in the samples, and their dependence on inter-grain coupling. Such measurement technique results in a FORC-distribution [39] that can be used to express the spreading of the irreversible magnetisation reversal mechanisms taking place in the sample, as a function of local coercive field H_C_ values and mutual magnetic interactions among grains, expressed in terms of a so-called bias field H_B_. Therefore, the peak of the FORC distributions measured for all the studied samples will be analysed to identify the spreading of local coercive field values, due to the grain size distribution in the sample, and the presence of inter-grain interactions affecting the magnetisation reversal processes.

All as-deposited samples were chemically treated for 5 h in a 2 molar aqueous solution of hydrochloric acid without applying any external potential and labelled S500_5h, S150_5h and S50_5h, respectively.

The morphological and surface transformations and the magnetic variations induced by the chemical treatment were characterised by means of the aforementioned experimental techniques.

## 3. Results and Discussion

**Figure 1:**
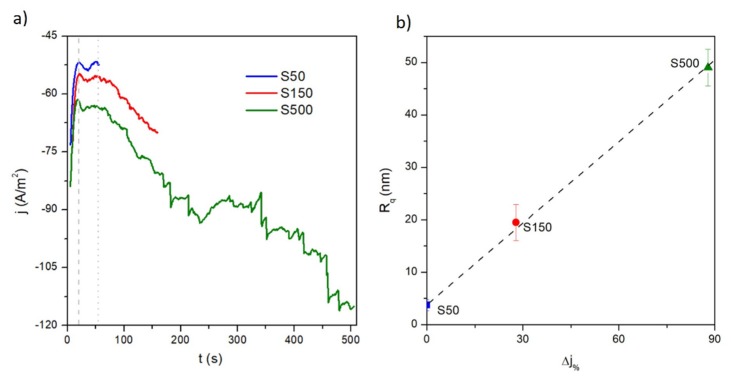
(**a**) Current density as a function of time recorded during the potentiostatic deposition of FePd thin film samples; (**b**) root mean square roughness (R_q_) as a function of variation of current density values  ∆j (see text for details) over the deposition time.

Current density (j) vs. time curves recorded during the potentiostatic deposition of FePd thin film samples are shown in Figure 1a. At the beginning of the electrodeposition process, the j(t) curves show a reduction of their absolute value reaching a minimum (|j|_min_) at around 20 s (indicated by the grey vertical dashed line in Figure 1a); this first portion of curve corresponds to the accumulation of a capacitive charge at the interface between the electrode and the solution [40]. Later, the FePd deposition occurs at an increasingly negative current density, which stays approximately constant only in the time interval 25–60 s (time interval between the grey vertical dashed and dotted lines in Figure 1a).

After that, the strong increase in |j|(t) over time, observed for the deposition of S150 and S500 samples, indicates that the deposition conditions are changing and that the surface area of the working electrode is increasing [40,41]. As a consequence, the formation and growth of an increasingly rough film over deposition time is expected.

**Figure 2:**
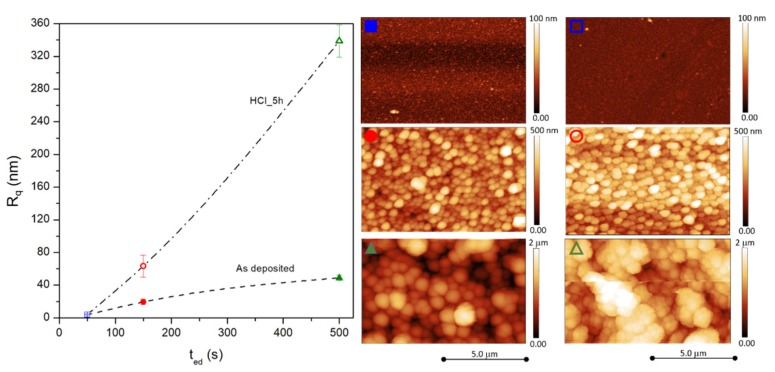
Root mean square roughness (Rq) for as-deposited and chemical etched samples as a function of electrodeposition time. At the right, the corresponding AFM images, identified by the same symbols and colours used in the graph.

This hypothesis is corroborated by the results of surface analysis of the samples obtained by the AFM; representative images for all samples are reported in Figure 2. Indeed, the root-mean-square roughness (R_q_) values measured from the AFM images turn out to monotonically increase as a function of electrodeposition time.

In addition, the R_q_ increase is also linearly correlated with the $∆j=\left|\left(j_{stop}-j_{min}\right)/j\right|$ parameter (see Figure 1b), which takes into account the variation of current density values over the deposition time; $j_{\mathrm{stop}}$ is the current density value at which the electrodeposition process is stopped. This correlation indicates that the progressive increase of working electrode surface area is developed by means of an increasing of surface roughness. This behaviour makes the growth in thickness nonlinear as a function of the deposition time.

**Figure 3:**
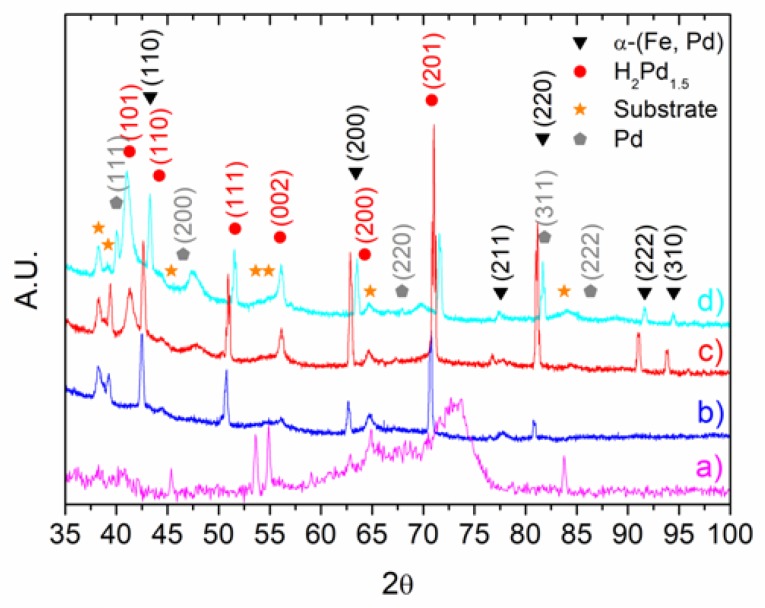
Grazing angle XRD pattern of: (a) substrate and electrodeposited FePd thin films (b) S50, (c) S150, (d) S500.

The grazing angle patterns of electrodeposited FePd thin films are reported in Figure 3. The contribution of the substrate is evident in all samples, independent of their thickness. Reflections peaks, labelled as black triangles in Figure 3, are associated to an α-(Fe, Pd) solid solution phase: however their position is shifted to lower angles at decreasing deposition time, meaning that a variation of stoichiometry and a different ratio of deposition are taking place. This issue, already reported in literature [42], is due to a local increase of pH of the near-electrode layer, that can cause the precipitation of iron hydroxide at quite negative potential, when the electrodeposition bath does not contain complexing agents: in this work, the addition of sulfosalicilic acid and Fe^3+^ ions as an iron source instead of Fe^2+^ avoids iron hydroxide precipitation [29] but not the local pH increase; as a consequence, a change in the pH occurs. Furthermore, the hydrogen evolution at the sample surface favours the inclusion of hydrogen into the deposit [42].

When deposition is performed for longer times (sample S500, cyan pattern in Figure 3), the fluctuation of the electrolyte composition in proximity of the working electrode becomes stronger, causing the deposition of a further phase identified as almost pure palladium [43].

**Figure 4:**
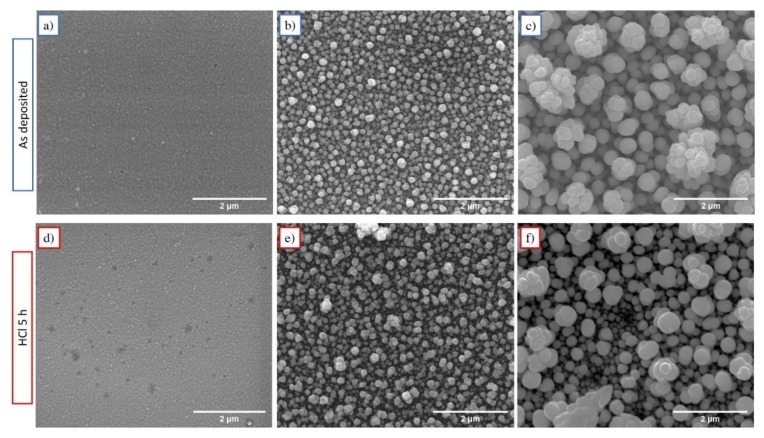
SEM images of as-deposited (**a**) S50, (**b**) S150, (**c**) S500 samples and etched (**d**) S50_5h, (**e**) S150_5h, (**f**) S500_5h samples in a 2 M aqueous solution of HCl for 5 h.

A significant amount of a tetragonal Pd_1.5_H_2_ phase appears in all samples due to the high affinity between hydrogen and palladium [44,45]. The formation of this metal hydride usually induces a lattice distortion followed by surface cracking [46]; however, SEM images reported in Figure 4 do not show this evidence.

A shift of reflections of the Pd_1.5_H_2_ phase is observed on decreasing the time of deposition. This is possibly due to change in stoichiometry of the hydride phase as a consequence of the increase of pH and, therefore, the amount of Pd electrodeposited.

The ratio of Fe:Pd was determined using EDS resulting in Fe_68_Pd_32_, Fe_66_Pd_34_ and Fe_69_Pd_31_ for S50, S150 and S500 sample, respectively. However, these stoichiometry values of as-deposited FePd alloy are affected by a high error because the EDS technique is not able to detect the H atoms present in the samples (Pd_1.5_H_2_ phase observed by XRD) and, as a consequence, it is not possible to distinguish the correct fraction of the Pd atoms present in the FePd alloy from those in the Pd_1.5_H_2_ metal hydride. Therefore, these EDS data are only used as a reference value to obtain the variation of Fe content in each sample after the chemical etching, as discussed in the following.

If the R_q_ value (shown in Figure 2) mainly provides information on the change of the morphology along the vertical direction, the lateral growth of the grains as a function of electrodeposition time was studied by means of SEM measurements.

**Figure 5:**
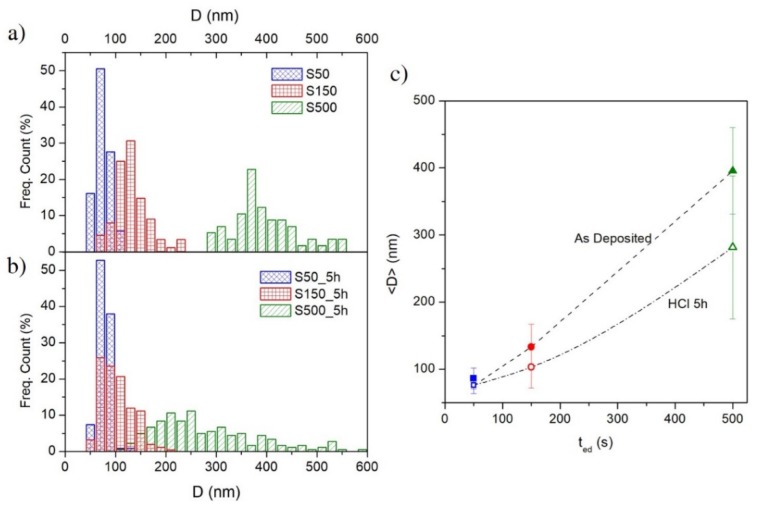
Grain size distributions of: (**a**) as-deposited samples and (**b**) etched samples in a 2 M aqueous solution of HCl for 5 h; (**c**) grain mean diameter < D > evolution as a function of electrodeposition time.

The surface of electrodeposited samples shows a rough granular morphology constituted by spherically shaped grains with different size as a function of deposition time (see Figure 4a–c). In particular, the grain size of FePd thin films is observed to increase significantly as a function of deposition time. In the early stages of the deposition, several nucleation centres on the Au substrate are localized and, for short deposition time intervals, the grains do not have a chance to grow and the film appears dense (see Figure 4a). As a consequence, the S50 sample displays small grains uniformly distributed over the substrate surface characterised by a narrow size distribution (Figure 5a) with a mean value < D > ≈ 85 nm, as shown in Figure 5c.

On increasing the deposition time, the grains are able to grow in spherical shape and their size distribution becomes wider (see Figure 5a) and the mean value increases to < D > ≈ 133 and 395 nm for the S150 and S500 sample, respectively, as shown in Figure 5c. The observed simultaneous increase of surface roughness and of the grain size indicates a 3D growth in the films as a function of the electrodeposition time, and is probably related, as already mentioned above, to the current density variation j(t) during the potentiostatic electrodeposition. The |j(t)| increase over the deposition time (see Figure 1a) promotes an increasingly rapid growth of independent grains over the coalescence growth mechanism by inhibiting a compliant film deposition.

The electrodeposited FePd alloy samples were chemically treated for 5 h in a 2 molar aqueous solution of HCl acid in order to induce a free corrosion of the sample surface promoting a variation of its morphological and physical properties.

**Figure 6:**
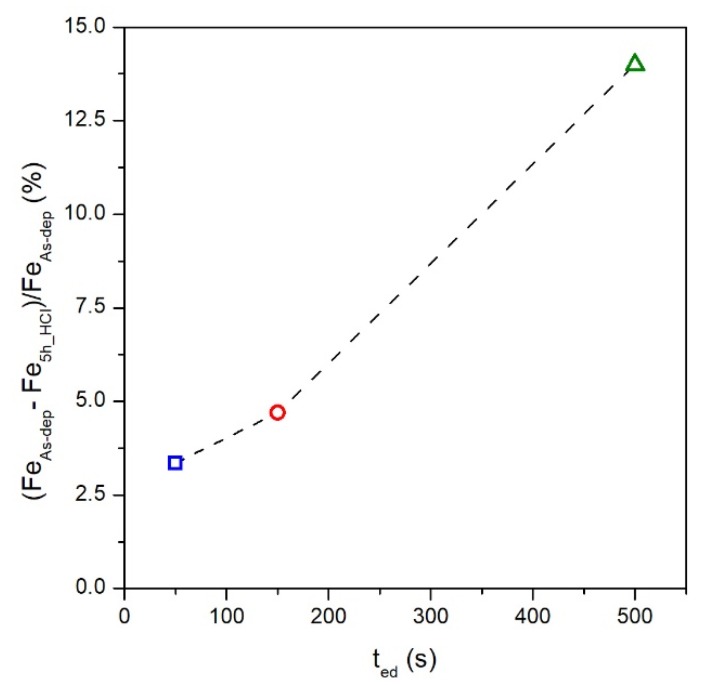
Percentage reduction of Fe contained in the FePd alloy before and after chemical etching as a function of the electrodeposition time.

The measured FePd alloy stoichiometry after the chemical treatment is Fe_66_Pd_34_, Fe_63_Pd_37_ and Fe_60_Pd_40_ for S50_5h, S150_5h and S500_5h sample, respectively. The observed variation of the stoichiometry with respect to the initial values indicates that a selective dissolution of the FePd alloy occurs in which the removal rate of the Fe atoms is higher than the one of the Pd atoms in all samples. This effect is similar to a dealloying process [18,47,48,49,50] in which the less noble element of the alloy, Fe in FePd alloy, is selectively removed by the 2M HCl solution. The percentage reduction of Fe contained in the FePd alloy before and after chemical etching for each sample is shown in Figure 6.

The Fe reduction rate is not constant among samples, but it monotonically increases as a function of the electrodeposition time. This effect can be ascribed mainly to the different fraction of sample surface area exposed to the aggressive 2 M HCl solution. The previous characterisations of R_q_ and < D > values indicate that the as-deposited S500 sample shows the biggest exposed surface area, in agreement with the highest Fe dissolution rate during the chemical process. On the other hand, the as-deposited S50 sample characterised by a low R_q_ and small grains size exposes a lower surface area to the acid solution making the chemical etching very slow.

The morphological transformations of the surface of FePd thin films induced by the chemical etching were characterised by the aforementioned AFM and SEM techniques. A primary consequence of the selective Fe dissolution is the increase of the average surface roughness (R_q_) and of the associated standard deviation. This effect is substantially clear for the S150_5h and S500_5h samples whose R_q_ values increase to 63 and 340 nm, respectively, as shown in the Figure 2. On the contrary, the S50_5h sample keeps almost the same roughness properties as the as-deposited S50 sample indicating a different response to the chemical etching.

In addition to the effect on surface roughness, also the grain size is affected by the chemical etching. SEM images in Figure 4d–f show that the arrangement of the spherical grains in S150_5h and S500_5h sample appear less dense with a slightly higher separation among them. No significant change is visible in the S50 sample, where the structure does not seem to be altered by treatment in HCl acid (see Figure 4a,d).

In addition, Figure 5b shows the variation of grain size distribution induced by the chemical treatments. The grain size distribution of the S500_5h sample is uniform over a much wider dimensional range than the one of as-deposited S500 sample and with an average grain size reduced to < D > ≈ 282 nm (see Figure 5c). In particular, this enlargement of the distribution induced by the chemical etching occurs completely towards smaller grain sizes while keeping the size of the larger grains unchanged. This change in the distribution shape indicates that the corrosion rate is not uniform on each grain but the as-deposited larger grains are more resistant. This evidence can be explained by assuming that the alloy stoichiometry of the as-deposited larger grains is richer in palladium or consists in pure palladium (as observed in XRD pattern), hindering the entire corrosion process.

The chemical treatment induces similar effects, but with less intensity, also on the grain size distribution of the S150_5h sample (see Figure 5b,c). The average size < D > is slightly reduced and the distribution becomes slightly more asymmetric towards lower size while maintaining a rather narrow peak and a values dispersion comparable to the ones of as deposited S150 sample. On the contrary, the effects induced by the same chemical etching on the grain size distribution of the S50_5h sample are marginal since the average (see Figure 5b,c), the dispersion of the values and the distribution are comparable to those of the as-deposited sample. In agreement to the EDS characterisation (see Figure 6), the topographic and morphological features of the etched samples surface confirm that the etching rate of the surface is strongly correlated with the surface area induced of the as-deposited samples.

**Figure 7:**
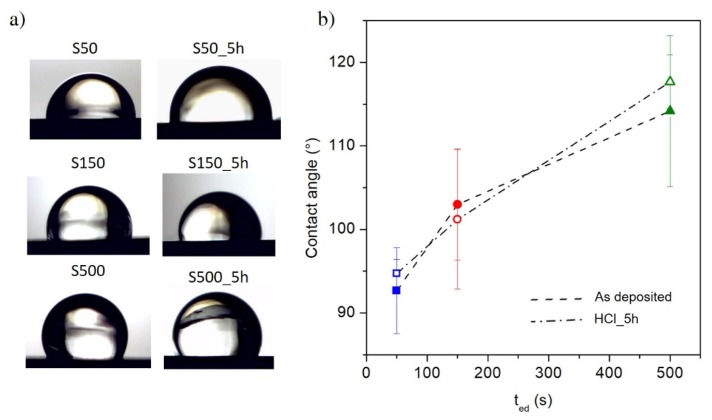
(**a**) Water drop on the samples surface; (**b**) contact angle evolution as a function of electrodeposition time for as-deposited and etched samples.

The surface morphology of the sample strongly influences its wettability properties [31,32,33,34,35,51] which are characterised by measuring the contact angle between a drop of water and the surface itself, see Figure 7a. The measured contact angle values are reported for all samples in Figure 7b where no significant differences are observed among as-deposited and etched samples. In particular, the contact angle values increase as a function of electrodeposition time indicating a more hydrophobic surface.

Several models describe the connection between the wettability behaviour of a surface and its roughness by means of some parameters calculated from 2D AFM profiles such as the mean slope of the roughness motifs (1/K_r_) and the measure of the steepness of the sample surface (R_q_) [30,31,32,33,34,35]. Both 1/K_r_ and R_q_/quantities, defined in the experimental section, include information both on the horizontal distance and on the vertical profile of the peaks in order to give an overall description of the contact surface on which the drop is released.

**Figure 8:**
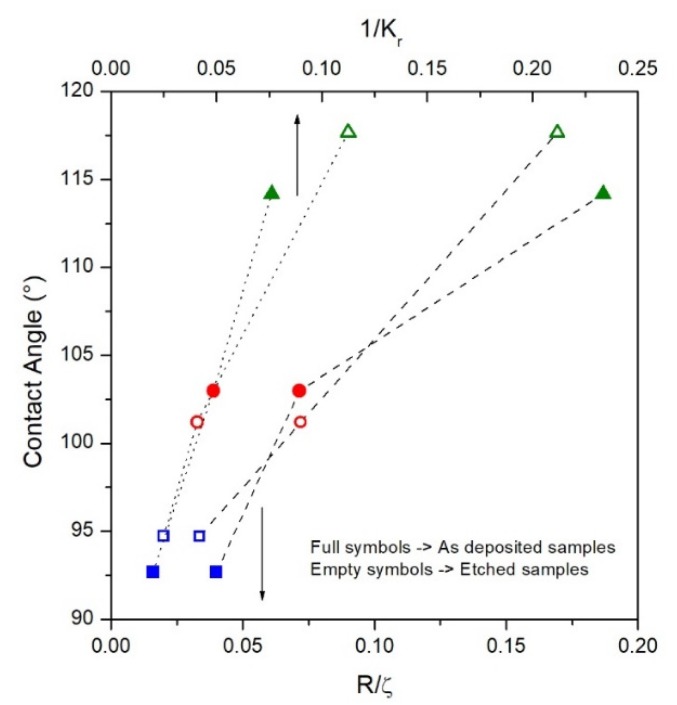
Contact angle values as a function of: 1/Kr parameter (dotted line) and R_q_/ξ parameter (dashed line) for as-deposited (full symbols) and etched (empty symbols) samples.

The 1/K_r_ and R_q_/parameters were calculated for all studied samples both before and after the etching treatment and their dependence on the measured contact angle is shown in Figure 8.

The 1/K_r_ and R/parameters monotonically increase as a function of contact angle and, therefore, as a function of the deposition time (see Figure 7b) for both as-deposited and etched samples. This development indicates a surface evolution over the deposition time, in which the vertical growth of the peaks (expressed by R and R_q_ for Kr and R_q_/ξ, respectively) is faster than the increase in their horizontal distance (expressed by AR and ξ for Kr and R_q_/ξ, respectively). This behaviour disadvantages the spreading penetration of the droplet into the sharp local irregularities on the surface, increasing the volume of air between the solid surface and the droplet [30]; as a consequence, the contact angle value increases. Therefore, the wettability of the FePd sample surface decreases as a function of deposition time becoming more hydrophobic (> 90°). Similar wettability properties of film surface are observed after the chemical etching.

**Figure 9:**
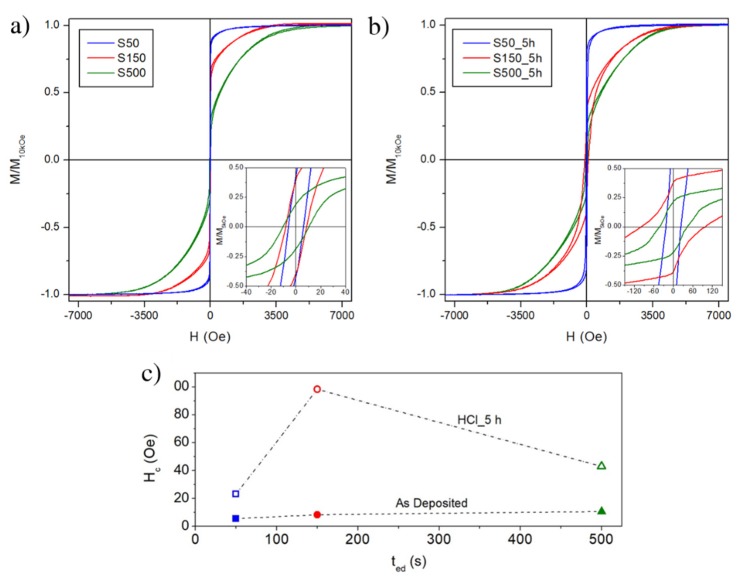
(**a**) Room temperature hysteresis loops of as-deposited samples; (**b**) room temperature hysteresis loops of etched samples in a 2 M aqueous solution of HCl for 5 h; (**c**) evolution of coercive field as a function of electrodeposition time for as-deposited and etched samples.

**Figure 10:**
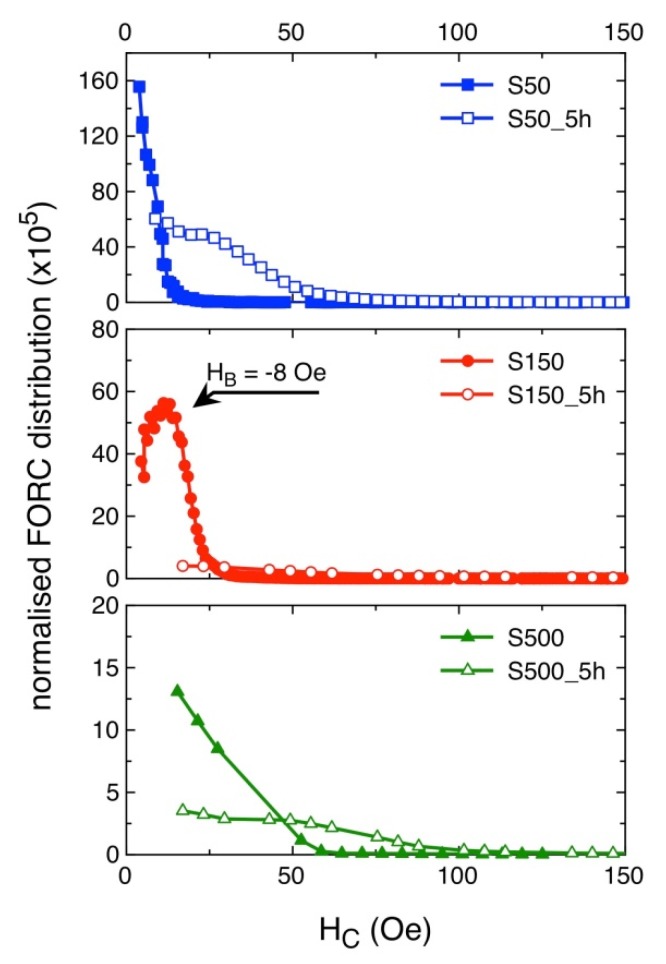
First Order Reversal Curves (FORC) distribution profiles as a function of the coercive field, taken at a bias field H_B_ = 0 for all samples, except S150. Full symbols correspond to as-deposited samples; the equivalent open symbols correspond to the samples submitted to chemical etching.

Room-temperature hysteresis loops of S50, S150 and S500 samples are shown in Figure 9. The magnetic field is applied in the FePd thin film plane and the curves are normalized to the magnetisation value at H = 10 kOe. The magnetisation reversal process of the S50 sample displays the typical features of a soft magnetic material [52]: a single irreversible magnetisation jump with a small coercive field (H_c_ ≈ 6.3 Oe). This is also evident by analysing the FORC distribution as a function of the coercive field, reported in Figure 10: the S50 sample is characterised by a sharp coercivity distribution at low fields. By increasing the deposition time, the magnetisation reversal as observed in the hysteresis loops of Figure 9a takes place on an increasingly wider magnetic field range; the single irreversible magnetisation jump reduces its intensity whereas a reversible mechanism becomes clearly visible with a slow approach of magnetisation to saturation. In addition, the coercive field slightly increases to 11.7 Oe for the S150 sample and to 23.7 Oe for the S500 sample. This is reflected in the FORC distributions of Figure 10, whose intensities decrease as the deposition time increases, indicating a progressively reduced contribution of the irreversible processes to the magnetisation reversal, at the advantage of reversible rotation mechanisms, which do not contribute to the FORC curves intensity. At the same time, the FORC distribution becomes wider as a function of the coercive field as the deposition time increases, in agreement with hysteresis loops.

This evolution of magnetic properties is fully compatible with the morphology changes observed by AFM and SEM characterisation and previously discussed. In the case of the S500 sample, the large grain size and the consequent high roughness imply a dominant role of the grain shape and crystal anisotropy in orienting the magnetisation. Therefore, at remanence the magnetisation is mostly randomly oriented in space, following the local easy axes of individual grains; upon increasing the applied field, reversible rotation processes bring the sample magnetisation to saturation. The same picture holds for the S150 sample, where, however, the small grain size and roughness are responsible for a smaller shape and crystal anisotropy energies; as a consequence, the reversible rotation processes affect a smaller fraction of the total magnetisation and the saturation is approached in a narrower field range. The S50 sample, instead, is characterised by smaller grain size and roughness, and a more compact morphology; in this case, inter-grain interactions overcome the single-grain shape and crystal anisotropy energies, inducing the formation of domain structures that cover several grains; their anisotropies are then largely averaged out, resulting in a magnetically soft behaviour with a fast approach to saturation determined by the absence of significant local anisotropies hindering the domain wall motion and forcing magnetisation rotation.

The magnetic behaviour above described is isotropic in the film plane for all the as-deposited samples as confirmed by the prefect superposition of the hysteresis loops measured in two in-plane orthogonal direction, see Figure S1 in the Supplementary Materials.

Once the samples are submitted to chemical etching, some important changes in the hysteresis loops take place, as shown in Figure 9b, as well as in FORC distributions vs. coercivity, depicted in Figure 10. Overall, the sample deposited for 50 s remains the softer and with the steeper approach to saturation, whereas for the other two samples the contribution of magnetisation rotation toward saturation seems to have increased even further. Indeed, while for all three deposition times the FORC distribution has a lower intensity after HCl etching, the decrease is way more pronounced for the S150_5h and S500_5h samples with respect to the S50_h one, indicating, for them, a predominant role of reversible rotation processes in the magnetisation reversal. However, the most significant change with respect to the non-etched samples is the large variation of coercive field, summarised in Figure 9c. As discussed earlier, the effect of the chemical etching process is to reduce and spread the grains size, make the grains more far apart, and increase the roughness. This is reflected by the FORC distribution of the three etched samples, that with respect to their as-deposited counterparts display a much larger spreading of coercive field values, that are due to the increased size distribution of the grains induced by exposition to HCl. As the grains are also more far apart, they also become more magnetically decoupled; as a result, their reciprocal interactions become weaker with respect to the as-deposited films. For the S50_5h sample this means that the mechanism of local averaging out of the magnetic anisotropy is less efficient, and the coercive field increases. However, inter-grains coupling is still largely present, due to the more compact morphology of this sample, that is little affected by the chemical etching. As a consequence, the hysteresis loop still displays a fast approach to saturation after the coercive field has been overcome, with just a slightly more pronounced contribution of magnetisation rotation toward saturation.

At the opposite end, the S500_5h sample incurs in a similar evolution with respect to its as-deposited counterpart: the coercive field increases, and the loop branches remain separated over a larger field interval, indicating that irreversible processes are spread over a larger portion of the hysteresis loop, in agreement with the much wider grains size distribution measured for this sample.

The effects of the chemical etching on the magnetic properties are particularly evident in the S150_5h sample, displaying the largest coercivity value and the most significant change of loop and FORC distribution shape with respect to the as-deposited specimen. While grains borders etching, spreading of grains size, and their magnetic decoupling are still responsible for the observed variations of loop shape and coercive field values, in the S150_5h sample their effects are enhanced. Indeed, while for the 50 s deposition time the etching process is not sufficient to decouple the grains, and for the 500 s deposition time they were already largely independent in the as-prepared state, the specimen deposited for 150 s incurs in a more complex evolution: in its as-prepared state, and differently from all the other samples, the FORC distribution as a function of coercivity has its peak at a bias field of -8 Oe, as indicated in Figure 10. The presence of such a bias field indicates that the S150 sample is in a state where the dominant ferromagnetic behaviour is attributed to the magnetic properties of the individual grains plus the effects of their mutual interactions; conversely, the S50 sample magnetic properties are attributed to a collective ferromagnetic behaviour of the grains, that lose their independence in favour of an averaging out of the local anisotropies, whereas for the S500 one the magnetic properties are mostly due to the sum of those of the individual grains. After chemical etching, the significant magnetic decoupling of the grains occurring in the S150_5h sample induces the disappearance of the bias field in the peak of the FORC distribution, that goes back to H_B_ = 0 Oe, and the particularly enhanced changes of loop and FORC distribution shapes.

After the chemical etching, the isotropic magnetic behaviour in the film plane of all sample is preserved, see Figure S1 in the Supplementary Materials.

## 4. Conclusions

Potentiostatically electrodeposited FePd alloy thin films are characterised by different morphological, structural and magnetic properties as a function of electrodeposition time.

In addition to the desired α-(Fe, Pd) solid solution phase, XRD structural analysis reveals the formation of a pure palladium phase in the S500 sample and a tetragonal Pd_1.5_H_2_ phase in all samples due to the high affinity between hydrogen and palladium.

The increase of |j|(t) over deposition time indicates a progressive increase of surface area of the working electrode with direct and strong influence on the surface roughness and the grain size distribution of the FePd thin film. The FePd film surface appears dense and flat with small and uniformly distributed grains for deposition time of about 50 s; for longer deposition time, an increasingly rapid growth of independent spherical grains is observed favouring a reduction of film density with a strong increase of surface roughness and grain size.

Magnetic properties are fully compatible with the structural and morphological properties of the as-deposited samples. Soft magnetic features of hysteresis loop with a single irreversible magnetisation jump and small coercive field are observed in S50 sample indicating that the magnetic anisotropy on the film plane can be easily overcome. For longer deposition times, the increase of the roughness and of the grain size strongly affects the magnetisation processes in which reversible rotations appear to reach the magnetic saturation.

As-deposited samples show different response to chemical treatment (5 h in a 2 M aqueous solution of HCl). In general, the observed variation of the Fe:Pd ratio induced by the chemical etching indicates that a selective dissolution of Fe in the FePd alloy occurs in which the removal rate of the Fe atoms is higher than the one of the Pd atoms in all samples. However, differences arise among the different samples.

The flat and homogeneous surface of the S50 sample allows slow chemical etching and, as consequence, the roughness and the average grain size keep almost the same values after the treatment. On the contrary, the chemical etching is more efficient on the S500 sample surface characterised by the biggest exposed surface area. In this case, a huge increase of the surface roughness and a remarkable reduction of the grain size is observed with a slightly higher reciprocal distance; however, the corrosion rate is not uniform on each grain but the as-deposited larger grains appear more corrosion resistant. The most significant change in the magnetic properties of the etched samples with respect to the non-etched ones is the large increase of coercive field. This effect is due to the morphological evolution of grains that induce weaker inter-grain interactions with respect to the as-deposited films. Single-grain anisotropy (both shape and crystal) becomes more important over collective behaviour in the etched samples, thanks to the magnetic decoupling of the grains, that is particularly evident in the S150_5h sample. Even though the in-plane isotropic behaviour of all samples is not modified by the chemical treatment, etched samples develop a magnetic behaviour more dominated by single-grain properties, which results in an increased coercivity and a reduction of the irreversible contributions to the magnetisation reversal, in favour of reversible rotations.

A hydrophobic behaviour of the surface is observed in all FePd thin films. As a function of electrodeposition time, the 1/K_r_ and R/ parameters indicate that the vertical growth of the surface peaks develops faster than the increase in their horizontal distance disadvantaging the spreading penetration of the water drop into the sharp local irregularities on the surface and, as a consequence, the contact angle value is observed to increase. No significant differences on the wettability properties of film surface are observed after the chemical etching.

In conclusion, this work provides a comprehensive characterisation of the fundamental properties of these FePd metallic thin films and demonstrates the ability to fine tune these properties opening the way to further advanced studies focused on their possible use as catalysts with improved features of activity, selectivity and stability combined to the possibility to recover and reuse them thanks to their specific magnetic properties.

## Supplementary Materials

The following are available online at https://www.mdpi.com/1996-1944/13/6/1454/s1, Figure S1: (a) Room temperature hysteresis loops of as-deposited samples; (b) room temperature hysteresis loops of etched samples in a 2 M aqueous solution of HCl for 5 h. 0° and 90° labels indicate the two orthogonal in-plane directions along which the hysteresis loops are measured, Figure S2: FM profiles of the step height between the substrate and the electrodeposited S500, S150 and S50 samples.
