# Supplementary material for: Structural and Magnetic Properties of FePd Thin Film Synthesized by Electrodeposition Method
Source: Materials (Basel). 2020 Mar 23;13(6):1454. doi: 10.3390/ma13061454 (PMC7142880; doi:10.3390/ma13061454)
Supplement: Supplementary file 1 [file materials-13-01454-s001.pdf]

materials

Supplementary Materials

Structural and magnetic properties of FePd thin film synthesized by electrodeposition method

Gabriele Barrera [1,*], Federico Scaglione [2], Matteo Cialone [2,3], Federica Celegato [1], Marco Coïsson [1], Paola Rizzi [2] and Paola Tiberto [1]

[1] Istituto Nazionale di Ricerca Metrologica (INRiM), Advanced Materials Metrology and Life Sciences, Strada delle Cacce 91, I-10135 Torino, Italy; f.celegato@inrim.it (F.C.); m.coisson@inrim.it (M.C.); p.tiberto@inrim.it (P.T.)
[2] Dipartimento di Chimica e Centro Interdipartimentale NIS (Nanostructured Surfaces and Interfaces), University of Turin, Via Pietro Giuria 7, I-10125 Torino, Italy; federico.scaglione@unito.it (F.S.); matteo.cialone@uab.cat (M.C.); paola.rizzi@unito.it (P.R.)
[3] Departament de Física, Universitat Autònoma de Barcelona, 08193 Cerdanyola del Vallès, Spain
* Correspondence: g.barrera@inrim.it

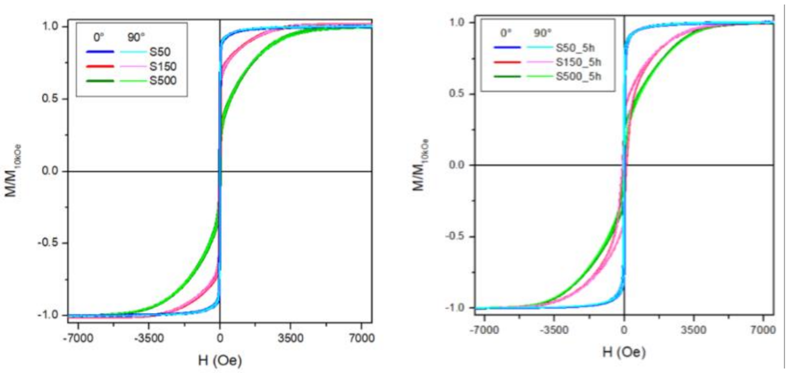

a) **b**)

Figure S1. a) Room temperature hysteresis loops of as-deposited samples; **b**) room temperature hysteresis loops of etched samples in a 2 M aqueous solution of HCl for 5 h. 0° and 90° labels indicate the two orthogonal in-plane directions along which the hysteresis loops are measured.

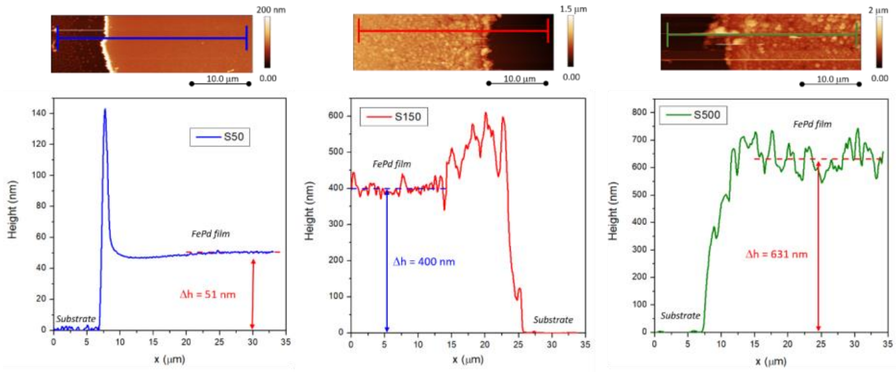

Figure S2. AFM profiles of the step height between the substrate and the electrodeposited S500, S150 and S50 samples.

Commented [M1]: There is no explanation for a), b) and c) in the figure.

© 2020 by the authors. Submitted for possible open access publication under the terms and conditions of the Creative Commons Attribution (CC BY) license (http://creativecommons.org/licenses/by/4.0/).
